# A Multi-Branch Training Strategy for Enhancing Neighborhood Signals in GNNs for Community Detection

**DOI:** 10.3390/e28010046

**Published:** 2025-12-30

**Authors:** Yuning Guo, Qiang Wu, Linyuan Lü

**Affiliations:** 1 Institute of Fundamental and Frontier Sciences, University of Electronic Science and Technology of China, Chengdu 611731, China; yn_guo@std.uestc.edu.cn; 2 Collaborative Innovation Center for Western Ecological Safety, Lanzhou University, Lanzhou 730000, China; 3 School of Cyber Science and Technology, University of Science and Technology of China, Hefei 230026, China

**Keywords:** community detection, neighborhood information, multi-branch, gradient modulation

## Abstract

The tasks of community detection in complex networks have garnered increasing attention from researchers. Concurrently, with the emergence of graph neural networks (GNNs), these models have rapidly become the mainstream approach for solving this task. However, GNNs frequently encounter the Laplacian oversmoothing problem, which dilutes the crucial neighborhood signals essential for community identification. These signals, particularly those from first-order neighbors, are the core source information defining community structure and identity. To address this contradiction, this paper proposes a novel training strategy focused on strengthening these key local signals. We design a multi-branch learning structure that injects a gradient into the GNN layer during backpropagation. This gradient is then modulated by the GNN’s native message-passing path, precisely supplementing the parameters of the initial layers with first-order topological information. Based on this, we construct the network structure-informed GNN (NIGNN). A large number of experiments show that the proposed method achieves a 0.6–3.6% improvement in multiple indicators compared with the basic model in the community detection task, and performs well in the t-test. The framework has good general applicability and can be effectively applied to GCN, GAT, and GraphSAGE architectures, and shows strong robustness in networks with incomplete information. This work offers a novel solution for effectively preserving core local information in deep GNNs.

## 1. Introduction

Complex networks are ubiquitous in diverse domains such as sociology, biology, and information science, with their community structure representing one of the most salient topological properties. A community is formally defined as a subgraph wherein nodes are densely intra-connected, while connections to the rest of the network are comparatively sparse [1]. The detection of these communities is fundamental to elucidating network organization and function, as well as modeling dynamic processes like information diffusion [2]. Consequently, this analytical process is instrumental in a wide array of downstream tasks, including recommendation systems, targeted marketing, load balancing, and the organization and visualization of complex data. In recent years, the field of community detection in networks has seen significant advancements. Traditional methods, such as modularity maximization [3] and spectral clustering [4], typically fall under the unsupervised category. However, their performance often degrades when applied to large-scale, sparse, or noisy networks [5].

With the rise of graph neural networks (GNNs), deep learning-based approaches to community detection have garnered considerable attention. A particularly effective strategy is to frame community detection as a node classification task, which leverages the powerful feature learning capabilities of GNNs [6]. In many real-world applications, we have access to the community affiliations of a small subset of nodes. For example, in a citation network, the research fields of some papers might be known. This limited amount of ground-truth information can be highly beneficial for improving community division. Message-passing GNNs have proven to be a robust and widely used classic model for semi-supervised node classification on graphs [7]. They serve as essential building blocks for many more advanced architectures, making their improvement a crucial area of research.

For an established network, the central challenge of community detection is to effectively distinguish signals from noise. For deep learning-based community detection methods, a common practice is to stack multiple layers of graph neural networks (GNNs). This approach aggregates node information from more distant neighbors, providing a broader, more global context that can effectively improve GNN performance [8]. However, this stacking of multiple layers also introduces the challenge of Laplacian oversmoothing, which causes the feature representations of all nodes to become increasingly close to each other and indistinguishable [9].

Prioritizing the aggregation of first-order neighbor information is a potential way to achieve this, as it maximizes high-fidelity signals while minimizing inter-community noise. The effectiveness of this approach is supported by multiple theoretical perspectives.

From the network structure and sociology perspectives, principles such as high aggregation, strong ties [10], and homophily [11] ensure a high degree of topological and attribute consistency among first-order neighbors, making them the most reliable source of information for community affiliation. From an information theory perspective, first-order neighbors provide the maximum signal-to-noise ratio (SNR) for community signals, whereas higher-order aggregation introduces cumulative noise through cross-boundary weak ties [10]. Assuming a homophily ratio h∈(0.5,1], the SNR for a *k*-hop neighborhood can be modeled as follows: $SNR^{\left(k\right)}\approx \frac{h^{k}}{1-h^{k}}$. As signal fidelity $h^{k}$ decays exponentially with distance *k*, the first-order neighborhood (k=1) inherently preserves the highest-fidelity structural information. In contrast, deep aggregation facilitates Laplacian oversmoothing, which homogenizes node representations and undermines their distinguishability. From the computational models’ perspectives, in modern architectures like graph neural networks (GNNs), excessive aggregation of long-distance information can lead to the “oversmoothing” [9] problem, which undermines the distinguishability of nodes. Therefore, from the standpoints of information fidelity, network microstructure, and the inherent limitations of computational models, focusing on low-order—and especially first-order—graph structural information [12] is a direct and reliable approach to community detection.

Enhancing homogeneity brings a decrease in global discrimination, which may seem contradictory to mitigating oversmoothing. However, the local structure information introduced from the first-order neighbor information can reduce the high-frequency noise and enhance the understanding of the local topology of the graph neural network, which is crucial for community detection. Therefore, it is necessary to design an appropriate algorithm structure to balance these two points, properly reducing the global discrimination, but effectively reducing high-frequency noise, which is also effective for improving the performance of community detection, and is also an effective way to alleviate the oversmoothing phenomenon.

For graph neural networks with multiple stacked layers, during backpropagation, the gradient is passed layer by layer from the final output. The gradients received by a preceding layer are already a mixture of information aggregated from subsequent layers via the graph structure. As a result, when these gradients are further aggregated by the preceding layer, they become increasingly mixed with information from distant neighbors.

To address this, we propose a method to supply gradients that do not contain graph structure information to the earlier layers of a multi-layer GNN. This ensures that the gradient aggregation at these initial layers captures only first-order neighbor information. By doing so, our approach effectively supplements the parameters of the earlier layers with essential first-order neighbor information.

This paper addresses the key challenge in multi-layer GNNs by considering the inherent graph-based message-passing path of each layer, which directly contains first-order neighbor information. We introduce a novel multi-path learning architecture, derived from message-passing graph neural networks (GNNs), to provide gradients during backpropagation.

We apply this architecture to the GCN [13], which possesses the most direct graph structure. This model, named the network structure-informed graph convolutional neural network (NIGCN), is designed to embed network structural information into the GNN’s parameters. Our experiments on multiple datasets and across various metrics demonstrate a significant improvement in its performance on both node classification and community detection tasks. We extend this approach to other message-passing GNNs, including GAT [14] and GraphSAGE [8], constructing the NIGAT and NIGraphSAGE networks, and find that our proposed structure remains effective. So in the experiments of this paper, the effectiveness of the auxiliary training branch is evaluated by comparing the performance improvement of the model with the auxiliary training branch to that of the base model. We propose that the underlying gradient modulation mechanism in this architecture effectively alleviates the Laplacian oversmoothing problem and enhances the model’s ability to utilize graph structural information [9].

Our main contributions include proposing the gradient modulation mechanism, presenting a multi-path learning architecture with general applicability, and conducting experimental verifications from multiple aspects.

The message-passing mechanism within the graph neural network’s gradient backpropagation path serves to embed network structural information directly into the model’s parameters. This approach enhances the model’s ability to leverage graph structure, thereby effectively alleviating the Laplacian oversmoothing problem. From the perspective of leveraging first-order neighbor information, we propose a multi-way learning structure based on the gradient modulation mechanism. An improved graph convolutional network [13] (GCN) architecture is proposed, which integrates a mechanism to embed network structural information directly into its parameters. The proposed model is named the network structure-informed graph convolutional neural network (NIGCN). This method has achieved a 0.6% to 3.6% improvement in multiple indicators compared to the basic model and has performed well in the t-test. We found that integrating it with various graph neural network (GNN) backbones, such as GAT [14] and GraphSAGE [8], and incorporating diverse types of auxiliary information consistently yields a positive impact on the performance of community detection. Furthermore, our findings indicate that this structure is also effective for inductive learning in dynamic networks and in networks with incomplete edge information. This shows the general applicability of the architecture.

The rest of this paper is organized as follows. Section 2 introduces the construction method, principle explanation, variant general applicability, and efficiency analysis of the multi-branch training strategy. Section 3 introduces the experimental content, including the experimental description, experimental results, general applicability experiment, ablation experiment, inductive learning, incomplete network, etc., and visually demonstrates the improvement of the model’s classification ability and the alleviation of the Laplacian smoothing phenomenon. Section 4 summarizes the work of this paper, explaining its strengths, weaknesses, and future improvement directions.

## 2. Methods

### 2.1. Semi-Supervised Modeling of Community Detection

This paper addresses the community detection task as a semi-supervised node classification problem on a graph. Given a graph G=(V,E), where *v* denotes the set of nodes and *E* is the set of edges, each node $v_{i}\in V$ is associated with an initial feature vector $x_{i}\in R^{d}$. We assume that a small subset of nodes, $V_{train}\subset V$, have known community labels $y_{i}\subset \left\{1,2,\ldots ,K\right\}$, where *K* represents the number of communities. Our objective is to learn a mapping function f:V→{1,2,…,K} that can accurately predict the community affiliation of all unlabeled nodes.

### 2.2. GNNs with Auxiliary Learning Branches

A key challenge in graph neural networks (GNNs) is Laplacian oversmoothing [9]. While GNNs aggregate multi-hop information to gain a broader receptive field, this smoothing operation causes node features to become increasingly homogenized as model depth increases. This oversmoothing feature severely restricts GNN performance on tasks like node classification. Therefore, a critical research question is how to preserve essential low-order information, especially within the GNN’s earlier layers. This focus is vital, as first-order neighbor information plays a core role in tasks like community detection, a significance supported by network structure, social network theory, and information theory, based on the research and theories previously discussed.

Therefore, this paper proposes a gradient modulation mechanism. This mechanism enables the backpropagation of gradients that do not carry graph structural information, thereby supplementing the earlier layers of a GNN with low-order neighbor information. This is achieved via the inherent message-passing pathway of the graph neural network itself. The core of this idea is to provide the initial GNN layers with stable and adjustable gradients during backpropagation. To ensure a high degree of learning and adjustment capability, these gradients must be non-linear.

To implement this, we designed an auxiliary learning branch [15] that uses a nonlinear structure of a linear layer → an activation layer → a linear layer to learn extra information. By training on stable input, this branch prevents the learning of overly irrelevant content, thus maintaining gradient stability. We use positive learnable parameters to control the magnitude of the transferred gradients. This branch is added to the backbone of a classic message-passing GNN, forming an improved network. Given its purpose of directly embedding network structural information into parameters, we name this model the NIGNN (network structure-informed graph neural network).

This paper first takes the most direct graph convolutional neural network [13] for message passing as the skeleton, and selects the two-layer network with the best empirical effect to add the branch structure mentioned above to the two-layer graph convolutional neural network.

We employ a two-layer GCN as the backbone. The propagation rule is defined as follows: First layer:

(1) $$H^{\left(1\right)}=dropout\left(\sigma \left(\hat{A}XW^{\left(0\right)}\right)\right)$$

Second layer:

(2) $$H^{\left(2\right)}=\hat{A}H^{\left(1\right)}W^{\left(1\right)}$$

The final classification output is obtained through a softmax layer:

(3) $$Z=\mathrm{softmax}(H^{\left(2\right)}W_{c})$$

Here, *X* is the node feature matrix, $\hat{A}$ is the normalized adjacency matrix with self-loops, σ(·) is a non-linear activation function (e.g., ReLU), and $W^{\left(0\right)}$, $W^{\left(1\right)}$, $W_{c}$ are learnable weight matrices. The loss function for the main task is the cross-entropy loss calculated over the labeled nodes $V_{train}$:

(4) $$L_{cls}=-\sum_{i\in V_{train}}y_{i}\log Z_{i}$$

We introduce an auxiliary learning branch from an intermediate layer, $H^{\left(1\right)}$, of the GNN backbone. This branch contains a lightweight prediction head $g_{\phi }\left(\;\cdot \;\right)$ designed to predict a predefined auxiliary objective.

(5) $${\hat{y}}_{aux}=g_{\phi }\left(H^{\left(1\right)}\right)$$

The loss function for the auxiliary branch, $L_{aux}$, is defined as the difference between the predicted value ${\hat{y}}_{aux}$ and the ground-truth auxiliary target $y_{aux}$.

In our experiments, we explore various auxiliary targets, including constant signals (e.g., all-zero or all-one vectors, and 0-1 interleaved signals) that contain no inherent graph structural information. We also utilize random signals, assigning a fixed random number to each node as an auxiliary target.

Our findings indicate that all these signals are effective and yield largely consistent results. This validates our core hypothesis—gradients that do not carry graph structural information can still supplement the GNN’s parameters with low-order neighbor information. This is achieved through the graph information transmission path of the message-passing mechanism itself during backpropagation.

Building on our core hypothesis, we further explore whether the choice of learning content affects performance. We propose that using first-order network structural information, specifically node degree, is a more effective learning signal. Our experiments (Section 3.4) confirm this: using node degree as an auxiliary learning target led to a slight performance improvement and, crucially, a more stable training effect. This finding provides further evidence for the theory we have put forward—that our method works by aggregating first-order neighbor information via the GNN’s native message-passing path during backpropagation. It reinforces our central argument that the primary driver of performance gain is the backpropagation process itself.

Because it contains learnable parameters, this network is vulnerable to overfitting. Consequently, its training necessitates an early stopping strategy. Overfitting arises because the new layers and parameters are continually optimized. Since the forward-propagated information is independent of the graph structure and the back-propagated information is low order, an excessive amount of this low-order information is fed back into the graph neural network’s parameters. This ultimately degrades the network’s capacity to recognize global information.

We attempt to address this with a regularization term. While it does alleviate overfitting and slightly improves community detection, it actually leads to worse results than without the term, given the support of an early stopping strategy. Our experimental results, which track predictive performance across multiple datasets over increasing epochs, show that without a regularization term, the peak value of the prediction index is higher. Furthermore, this high performance can be maintained for dozens of epochs during training and will not immediately decline after reaching the optimal level, making it easier to achieve the best results through an early stop strategy. Therefore, we chose not to add a regularization term in our model, except for specific datasets where it proved beneficial.

Further exploration reveals that incorporating a greater diversity of learning branches, each providing distinct learning content, and jointly conducting backpropagation can significantly enhance both classification performance and model stability. Accordingly, this paper adopts a multi-path learning strategy with three distinct content branches—node degree, a full 1s vector, and a 0-1 alternating vector. These branches are trained jointly through backpropagation.

**Figure 1:**
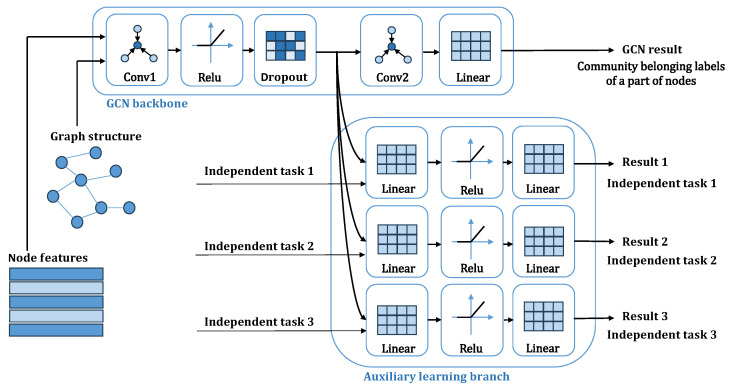
A complete NIGCN model with three auxiliary branches added. The upper part of the figure is the GCN backbone, and the learning results and community belonging labels of a part of the nodes calculate the classification loss function value. The lower part of the figure is the auxiliary learning branch, which calculates the loss function and provides the gradient by learning on different independent tasks.

In summary, this paper proposes a graph convolutional neural network model that embeds network structural information via a gradient modulation mechanism. This model, a direct extension of the previously mentioned NIGNN framework, is named the NIGCN model, as shown in Figure 1.

The complete NIGCN model consists of a two-layer graph convolutional network backbone and three additional learning paths corresponding to the degree, all-ones, and 0-1 alternating signals. The loss functions from these four paths are first scaled to a consistent order of magnitude using learnable parameters. After adding a regularization term, the combined loss is used for joint backpropagation, allowing all parameters to be collectively optimized.

### 2.3. Gradient Modulation Mechanism

Based on the preceding discussion, we propose the core hypothesis that in message-passing graph neural networks (GNNs), an auxiliary branch can inject additional first-order graph structural signals into the model. This is achieved through a gradient modulation mechanism that propagates gradient messages along the inherent message-passing path during backpropagation.

**Figure 2:**
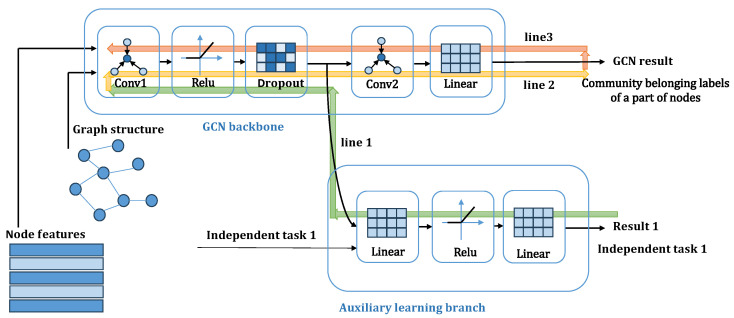
The parameter optimization path in the NIGCN model. Gradients learned for independent tasks are first backpropagated through green line1, then forward-propagated through yellow line2, then backpropagated through red line3, and finally propagated to all GNN layers.

The forward pass of our GCN backbone is defined as Equations (1) and (2). As shown in Figure 2, during each training iteration, the auxiliary branch (auxiliary learning branch) connects to the intermediate representation $H^{\left(1\right)}$. The gradient of the auxiliary loss, $L_{aux}$, with respect to the first-layer parameters, $W^{\left(0\right)}$, is calculated via the backpropagation path (green line):

(6) $$\frac{\partial L_{aux}}{\partial W^{\left(0\right)}}=\frac{\partial L_{aux}}{\partial H^{\left(1\right)}}\frac{\partial H^{\left(1\right)}}{\partial W^{\left(0\right)}}$$

The term $\frac{\partial H^{\left(1\right)}}{\partial W^{\left(0\right)}}$ in Equation (6) directly involves the normalized adjacency matrix $\hat{A}$ from the forward pass:

(7) $$\frac{\partial H^{\left(1\right)}}{\partial W^{\left(0\right)}}=\hat{A}X\circ dropout{\left(\sigma \left(\hat{A}XW^{\left(0\right)}\right)\right)}^{\prime }$$

Thus, the gradient message from the auxiliary branch, represented by $\frac{\partial L_{aux}}{\partial H^{\left(1\right)}}$, is explicitly modulated by the graph structure embedded within $\hat{A}$ as it propagates backward to update the first-layer weights $W^{\left(0\right)}$(represented by the green lines in Figure 2). This mechanism inherently injects graph structural information into the model’s parameters, even when the auxiliary signal itself (e.g., constant or random signals) contains no meaningful structural information. We believe this is the key reason why even simple, graph-agnostic signals can enhance performance.

During the next forward pass (yellow line in Figure 2), the model uses the updated parameters, which now carry these modulated structural signals. The resulting representations are used to calculate the main task loss, $L_{cls}$, which is then backpropagated through the entire network (red line in Figure 2). This final step refines all parameters of the GNN, effectively embedding the learned graph structure information throughout the model’s message-passing architecture.

The preceding text outlines the principle of our proposed method for embedding network structural information via backpropagation gradient modulation. In contrast to other graph neural network models that rely on auxiliary mechanisms like autoencoders [16] or random walks [17] to enhance graph structural information, our approach directly leverages the inherent message-passing paths and gradient backpropagation of GNNs. This makes our method more automated and inherently end-to-end.

### 2.4. Model Variants and General Applicability

Based on the preceding analysis, the proposed auxiliary learning branch is well-suited for message-passing graph neural networks (GNNs). Consequently, this paper extends the application of our method beyond GCNs to other message-passing GNN architectures, including the following:

Graph attention network (GAT) [14]: GAT assigns different weights to each neighbor via an attention mechanism. Since the resulting adjacency matrix from this mechanism still spans the entire graph, our auxiliary branches can also propagate gradients through this matrix, leading to performance enhancements.

GraphSAGE [8]: This model aggregates information through neighbor sampling, which restricts backpropagation to the sampled subgraph. Despite this limitation, experimental results demonstrate that our method still provides performance gains. This indicates that our approach can provide beneficial signals to the model through gradient modulation, even without relying on a complete adjacency matrix.

The auxiliary learning branch proposed in this paper demonstrates its generalizability by proving effective on two distinct GNN architectures—graph attention networks (GATs) and GraphSAGE.

Specifically, our method’s efficacy on GraphSAGE, a model that performs neighbor sampling and is well-suited for incomplete and dynamic graphs, indicates that our auxiliary branch is applicable to both inductive learning and transductive learning tasks. The ability to train our auxiliary branch using constant and random signals further ensures its applicability in scenarios where the complete graph structure is unknown, such as with dynamic graphs. This significantly broadens the application scope of GNNs integrated with our proposed structure.

### 2.5. Efficiency Analysis

The auxiliary branch only introduces a small prediction head, and it is introduced after encoding. The dimension is only related to the set encoding dimension. Therefore, the computational complexity of auxiliary branches is independent of the dimension of the input data and is linear in the number of nodes in the graph.

## 3. Experiments

### 3.1. Experiment Settings

The network of academic relationships has a distinct community nature. Cora, CiteSeer, and PubMed [18] are the most popular groups of paper citation networks used in community detection experiments as attributed networks, where nodes represent publications and links mean citations. The nodes are described by binary word vectors. The research directions of the papers are class labels. The size division of the training set, test set, and validation set is the same as that of Planetoid [18].

We also conducted experiments on two classic citation network benchmark datasets: Coauthor CS and Coauthor Physics [19]. Both datasets are derived from the Microsoft Academic Graph. In these networks, nodes represent authors, and edges denote co-authorship relationships. Node features are bag-of-words representations of paper keywords, while labels correspond to the authors’ research fields. Since there are generally few real labels in the community detection task, this paper chooses only 5% of the node community category labels that are known, of which 2.5% labels are used as the training set, 2.5% labels are used as the validation set, and the other 95% node community labels are used as the test set.

The basic statistics for these datasets are summarized in the Table 1.

To comprehensively evaluate the community detection performance of the NIGCN model, we employed a set of complementary evaluation metrics. Accuracy (ACC) directly measures the match between predicted and true labels. Normalized mutual information (NMI) quantifies the shared information between the two community partitions from an information-theoretic perspective, thus assessing structural similarity independent of specific label names. We also utilized two metrics based on node-pair analysis: the Adjusted Rand Index (ARI) [20], and the Jaccard index [21]. These methods assess whether the relationships between any two nodes in the predicted partition are consistent with those in the true partition. ARI, in particular, provides a more robust evaluation of clustering consistency by correcting for chance. Collectively, this group of metrics offers an objective and reliable quantitative assessment of the model’s performance from various perspectives.

To ensure the robustness and reproducibility of our experimental results, we adopted a consistent training strategy. All models were evaluated under the same data partitioning and training configuration. We used the Adam optimizer with a learning rate of 0.01 and an L2 regularization coefficient of 5e-4. The hidden layer dimension was set to 64. The models were trained for a maximum of 1000 epochs. We adopted an early stopping strategy to prevent overfitting. To avoid premature stopping due to random fluctuations in the initial training, a warm-up period was implemented. This strategy offers flexibility, as users can select either validation set accuracy or validation set loss as the monitoring metric, based on their specific needs. Validation set metrics are used to exclude cases of poor effect in the experiments. We calculate the mean and standard deviation on 50 independent runs of different random seeds.

To implement a multi-path learning approach, we introduced learnable parameters for each path in our model. These parameters were applied in a squared form to the corresponding calculated loss. To prevent over-optimization and the loss function from converging to zero, we employed a modified loss term in the form of ${\left(learnableparameter\right)}^{2}*\left(loss+1\right)$. This adjustment proved effective in improving performance across all three datasets.

Specifically for the Cora dataset, which has a small number of nodes and a low feature dimension, we found that using the inverse square of the learnable parameter yielded better results. Additionally, incorporating batch normalization [22] into the auxiliary learning branch helped mitigate overfitting.

The auxiliary training branch proposed in this paper is currently only used for semi-supervised community detection tasks, while most of the non-deep learning community detection algorithms mentioned above are unsupervised learning algorithms. Since the semi-supervised algorithm has more labels for some nodes than the unsupervised algorithm, the increase of information can naturally bring better results, so the experiment in this paper does not compare with the unsupervised learning algorithm.

For semi-supervised deep learning algorithms, the most classical models are GCN, GAT, and GraphSAGE [23], and most of the other models are improvements and variants of the three models. Therefore, the experiment of this paper will apply the auxiliary training branch proposed in this paper to these three models, and discuss the effectiveness of the branch by the improvement obtained after the application of the auxiliary training branch compared with the base model. Since the graph structure of the GCN model is the most direct and complete, we first focus on the effect on the GCN model.

Our models were trained on an NVIDIA GeForce RTX 4060 Ti GPU with 8 GB of memory, using PyTorch 2.4.1 and CUDA 13.0.

### 3.2. Experimental Results

We compared the NIGCN model and the GCN model with the added auxiliary learning branch to illustrate the effectiveness of the added structure proposed in this paper. The results are shown in the Table 2 and Table 3.

As shown in the tables, the NIGCN model, enhanced with the auxiliary branch structure, consistently achieves a significant improvement over the baseline GCN model on every dataset. This performance gain is evident in both overall clustering accuracy and the three metrics used to evaluate community division quality. To further validate the superiority of our method, we conducted independent t-tests between our model and the baseline. The results show that our improvements are statistically significant with *p*-values substantially less than 0.001 (p≪0.001) across all datasets and metrics.

From the results in the table, we can see that the improvement degree of the NIGCN model on the community detection indicators is generally greater than the classification accuracy of the overall indicators. This further reflects the idea that first-order neighbor information plays an important role in community structure discovery.

### 3.3. Apply to Other Basic Models

We extended our approach by adding a single-path auxiliary learning component to both the graph attention network (GAT) [14] and the unsampled GraphSAGE [8] models, maintaining the same hyperparameters as previously described. We used the Adam optimizer with a learning rate of 0.01, a hidden layer dimension of 64, a dropout rate of 0.5, and an L2 regularization coefficient of 5e-4. Models were trained for different epochs, and every epoch was trained for 100 seeds. For each dataset, we multiplied a tuning factor based on the initial training loss of the backbone and auxiliary branches to achieve unity of magnitude.

For intuitive demonstration, we only adopted the most common Cora, CiteSeer, and PubMed datasets, and only used accuracy as the metric.

**Figure 3:**
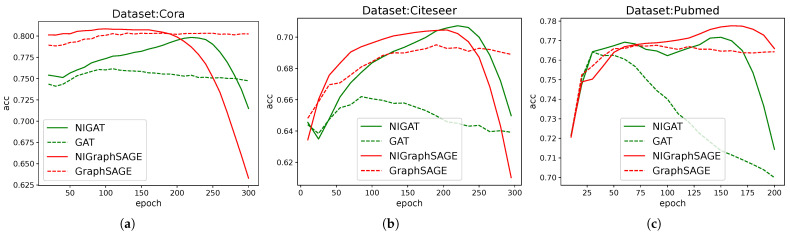
Comparison of GAT and GraphSAGE models with and without auxiliary learning branches. This figure shows the average accuracy changes of four models in the (**a**): Cora, (**b**): CiteSeer, and (**c**): PubMed datasets with 100 seeds under different training epochs. The solid line represents the model with the auxiliary learning branch added, the dotted line represents the original model without addition, the red line represents the GAT model, and the green line represents the GraphSAGE model.

To provide a clear and intuitive visualization, we plotted the change in accuracy over training epochs. The results are shown in Figure 3.

### 3.4. Ablation Study

**Figure 4:**
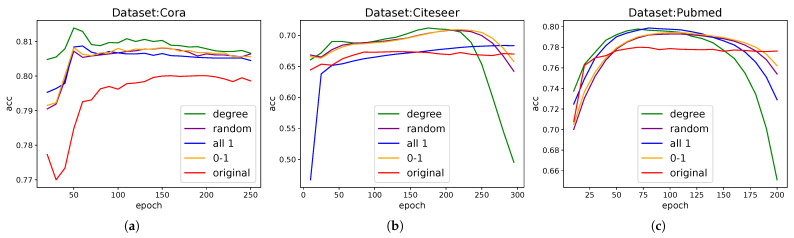
Ablation study with different auxiliary targets. Here is a comparison of the model with the four independent tasks versus the model without the auxiliary branch (red line). The results show the average accuracy changes of 100 seeds in the (**a**): Cora, (**b**): CiteSeer, and (**c**): PubMed datasets under different training epochs. The different colored lines shown in the figure represent different tasks.

Through a systematic ablation study, we compared the effects of different auxiliary targets on model performance. For fair comparison, each model uses the same hyperparameters except for the auxiliary branch, and the normalization operation is removed to investigate the processing ability of the original data and topology, so performance is reduced in some cases. Our findings are as follows, as Figure 4 shows:

- Node degree: Although our idea is to add gradients independent of the graph, it is also necessary to explore the effect of adding variables related to the first-order structural information of the graph, and we chose the degree. When a degree is used as an auxiliary target, the performance of the model is slightly improved, and the effect is more stable. It shows that the information related to the first-order neighbors of the graph is better for auxiliary learning, and the auxiliary learning branch extracts and complements the first-order information. Compared with other information, the improvement effect is small, which also indicates that the gradient modulation mechanism is mainly in play.
- All-zeros/zero-ones vectors: Even with a constant auxiliary target, we observed an improvement in model performance. This demonstrates the effect of the gradient modulation mechanism itself: when gradients are filtered and propagated through the adjacency matrix, they still positively impact model training.
- Random numbers: The use of fixed random numbers as an auxiliary target also resulted in performance gains, further validating our core explanation that gradients propagate through adjacency matrices.

The additional effects of the above information are almost the same, and all have a significant improvement in the GCN model; these results strongly support our central hypothesis—auxiliary branches can embed first-order neighbor information through gradient modulation to enhance model performance.

### 3.5. Inductive Learning

Inductive learning [8] is a machine learning paradigm that fundamentally contrasts with transductive learning [13,24]. At its core, inductive learning aims to build a model that can generalize. It does this by extracting generalizable rules from a given training dataset, which are then applied to make predictions on entirely new, unseen data without requiring model retraining.

In the context of graph neural networks (GNNs), this means a model is trained on a subset of labeled nodes and can then be used directly to predict the community labels of any new or previously unseen nodes. This approach is especially well-suited for dynamic networks because it learns broad, transferable rules by aggregating neighbor information rather than simply memorizing the identity of each individual node. Given that network communities are often in a state of continuous change, inductive learning is a particularly crucial approach for community detection tasks.

The auxiliary learning branch proposed in this paper is applicable to a typical inductive learning model—GraphSAGE with neighbor sampling. This model is particularly well-suited for graphs with incomplete and dynamic nodes, as it operates by adopting neighbor nodes and loading graph subsets in batches.

For our experiments, we set up a two-layer GraphSAGE architecture. The second-layer SAGEConv aggregated features by sampling up to two first-order neighbors for each central node. The first-layer SAGEConv then resampled up to three neighbors from each of these two nodes. The auxiliary learning branch used an all-ones signal. We used a batch size of 1024 for both training and testing. For each epoch, we ran 100 random seeds and averaged the results to ensure stability.

**Figure 5:**
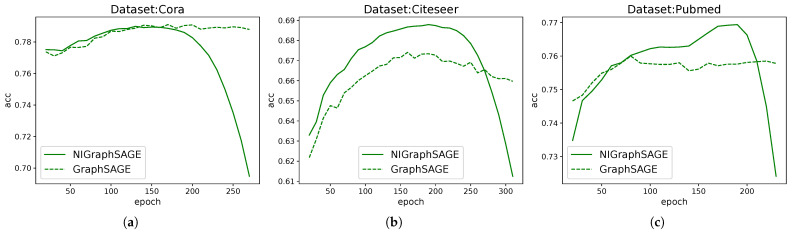
Inductive learning with graph node sampling. Here is a comparison of the GraphSAGE model with graph node sampling, with and without auxiliary branches. The results are the average accuracy changes of 100 seeds on (**a**): Cora, (**b**): CiteSeer, and (**c**): PubMed datasets under different training epochs. In the figure, the solid line represents the model with the added auxiliary learning branch using an all-ones signal, and the dashed line represents the original model without the addition.

Figure 5 illustrates our experimental results. The proposed auxiliary learning branch significantly improved performance on the CiteSeer and PubMed datasets, with even better results achieved by employing an early stopping strategy. While there was no significant improvement on the Cora dataset, performance did not degrade within the non-overfitting range.

Further testing revealed that even when a random signal was used for the auxiliary branch, performance gains could still be achieved. This finding corroborates our core hypothesis that performance is enhanced through gradient modulation rather than the specific learning content itself.

Crucially, because random and constant signals are independent of the graph structure and do not require knowledge of the complete graph, they can be effectively utilized in inductive learning scenarios.

### 3.6. Incomplete Network

In many practical applications, we often encounter incomplete network structures and missing topological information. For example, some edges may be missing in the Internet’s topology, and protein–protein interaction networks are frequently incomplete [25]. The absence of relational data is a common challenge during the data collection process. Consequently, a community detection algorithm’s sensitivity to information loss is a crucial factor to consider.

To evaluate this aspect, we randomly removed 10% and 50% of the edges from our datasets. With all other experimental settings remaining constant, we compared our single-path NIGCN model against the original GCN baseline. The results are presented in the figure below.

**Figure 6:**
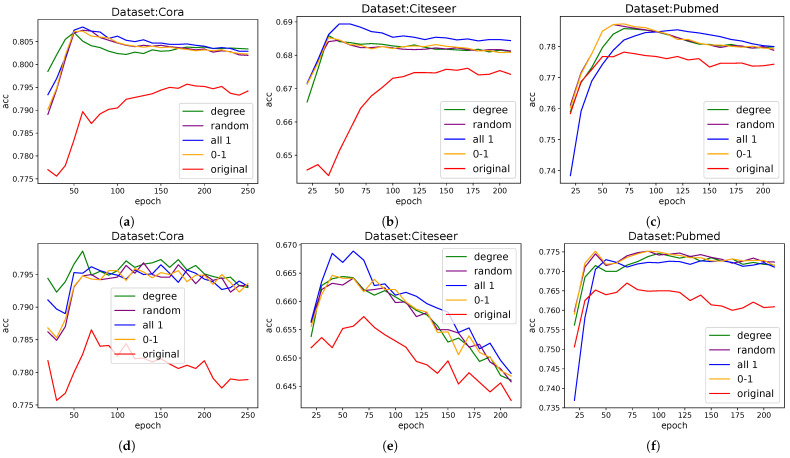
The case of missing edges. The graph compares the removal of a percentage of edges for the model with four independent tasks versus the model without auxiliary branches (red line). The results show the average accuracy changes of 100 seeds in the Cora, CiteSeer, and PubMed datasets under different training epochs. The different colored lines shown in the figure represent different tasks. The top three subfigures (**a**–**c**) show random removal of 10% edges, and the bottom three subfigures (**d**–**f**) show random removal of 50% edges.

As can be seen from Figure 6, the NIGCN model proposed in this paper still achieves effective improvement over the original model in the absence of edge data.

### 3.7. Representation Analysis and Limitations

t-SNE (t-distributed stochastic neighbor embedding) [26] is a powerful nonlinear dimensionality reduction technique primarily used to visualize high-dimensional datasets. Mapping data points into a two- or three-dimensional space allows humans to intuitively observe and understand complex data structures. Unlike traditional linear methods such as principal component analysis (PCA), t-SNE excels at preserving the local structure of the data. This allows it to reveal the intricate neighborhood relationships between data points in high-dimensional space, often presenting distinct cluster-like structures.

**Figure 7:**
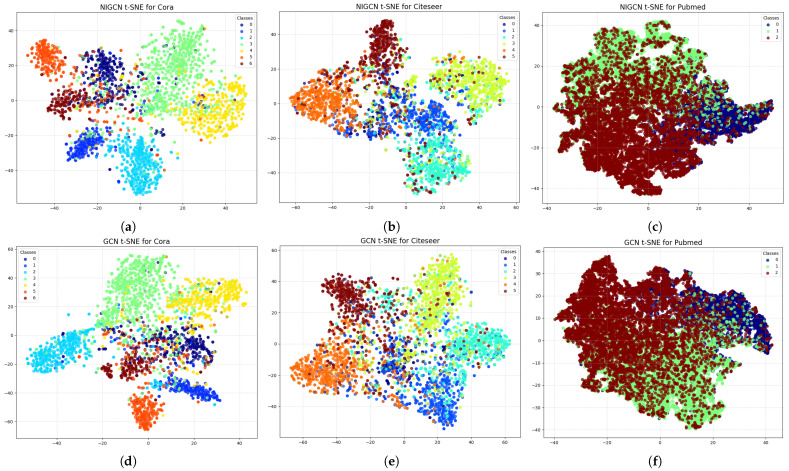
Interpret gradient modulation through the embedding vector-SNE. This figure shows the graph obtained by the t-SNE algorithm for the output encoding of the first graph neural network layer of the NIGCN model and GCN model on Cora, CiteSeer, and PubMed datasets. The top three subfigures (**a**–**c**) are the NIGCN model, and the bottom three subfigures (**d**–**f**) are the GCN model.

To visually demonstrate the effect of our proposed gradient modulation mechanism, we visualize the node embeddings from an intermediate layer of the NIGCN model in Figure 7—specifically, the output of the first GCN layer, where the auxiliary learning branch is introduced. A comparison between models with and without this branch reveals that the introduction of auxiliary branches results in greater separation and distinctness among nodes from different communities within the embedding space. This intuitively reflects the positive impact of gradient modulation on node representation learning.

Because communities are homogeneous, the proposed algorithm is effective in the community detection task. We also conduct semi-supervised graph node classification experiments on heterogeneous graphs, and the experimental results are not as good as the original GCN. We also compare the Dirichlet energy [27] of the NIGCN model and the GCN model in this paper, and find that the Dirichlet energy of the NIGCN model is lower than that of the original model in the training process, which means that the characteristics of adjacent nodes are more similar. So the method proposed in this paper mainly acts on homogeneous graphs.

## 4. Conclusions

### 4.1. Discussion

The auxiliary learning branch method presented in this paper has been validated across multiple datasets and models. The experimental results and subsequent mechanism analysis reveal several important insights:

- Primacy of First-Order Neighbors: Our results reaffirm the critical role of first-order neighbors, whose high-fidelity signal is central to community detection, a finding consistent with the efficacy of algorithms like LPA [28].
- Gradient as an Information Channel: The auxiliary branch succeeds even with arbitrary targets (e.g., random constants), proving that backpropagation acts as a vital, secondary channel for infusing graph structure. This gradient flow actively modulates backbone optimization, complementing forward-pass aggregation.
- Broad General Applicability: The paradigm demonstrates strong general applicability across diverse GNN architectures (GCN, GAT, GraphSAGE) and excels in challenging inductive settings, such as dynamic or incomplete graphs, by injecting structural information via the gradient pathway.
- Mitigation of Oversmoothing: t-SNE analysis confirms the auxiliary branch mitigates oversmoothing in deeper networks. This finding presents a novel, optimization-centric strategy to address this limitation, moving beyond purely architectural solutions.

Although the method proposed in this paper performs well on multiple datasets and models, it also has certain limitations:

- Limited Interpretability: The method provides a clear pathway for information to propagate back to the graph neural network layers through gradients. However, it lacks a specific theoretical explanation for how this transmitted information improves predictive performance. This paper does not delve into the underlying mechanisms from a formal perspective, such as through information theory or probability theory. This makes it challenging to fully understand the “why” behind the observed improvements.
- Overfitting and Practical Dependence: The network architecture is prone to overfitting. Its practical application is heavily reliant on an early stopping mechanism to prevent performance degradation on unseen data. While early stopping is a common technique, this dependency suggests the model may not be robust enough to generalize on its own, which could limit its real-world applicability without careful tuning and monitoring.

### 4.2. Conclusions and Future Work

This paper proposes a multi-path learning architecture for message-passing GNNs that integrates first-order structural information into model parameters through an auxiliary gradient mechanism. By implementing this architecture on GCN, GAT, and GraphSAGE, we introduce the NIGCN framework, which demonstrates robust performance across both transductive and inductive tasks. Furthermore, our proposed gradient modulation mechanism effectively mitigates the Laplacian oversmoothing problem. Experimental results on five benchmark datasets (Cora, CiteSeer, PubMed, Physics, and CS) confirm that our approach significantly outperforms baseline models in community detection tasks.

Future research will focus on (1) exploring adaptive auxiliary targets like node centrality; (2) integrating the architecture with self-supervised and contrastive learning; and (3) providing a rigorous theoretical analysis of the gradient modulation’s impact on oversmoothing. Overall, this work offers a novel training paradigm that enhances the structural awareness and performance of GNNs.

## Figures and Tables

**Table 1:** The following table is an introduction to the basic situation of the adopted data set. The four columns are the number of nodes in the dataset, the number of edges, the dimension of each node feature, and the number of communities (classes).

Dataset	Nodes	Edges	Features	Communities
Cora	2708	5429	1433	7
CiteSeer	3327	4732	3703	6
PubMed	19,717	44,338	500	3
Coauthor CS	18,333	81,894	6805	15
Coauthor Physics	34,493	247,962	8415	5

**Table 2:** Average performance in ACC and NMI metrics. The following table shows the comparison of ACC and NMI metrics on the model with and without the auxiliary learning branch. The results are the average and standard deviation of the results of the two models in independent experiments with 50 different seeds, and the part in bold is the effect of the proposed model.

	ACC		NMI	
	NIGCN	GCN	NIGCN	GCN
Cora	**0.8305 ± 0.0086**	0.8129 ± 0.0034	**0.6401 ± 0.0089**	0.6171 ± 0.0041
CiteSeer	**0.7146 ± 0.0039**	0.7063 ± 0.0025	**0.4483 ± 0.0054**	0.4124 ± 0.0019
PubMed	**0.7971 ± 0.0025**	0.7905 ± 0.0044	**0.3916 ± 0.0031**	0.3745 ± 0.0039
CS	**0.9133 ± 0.0029**	0.9030 ± 0.0030	**0.8249 ± 0.0033**	0.8096 ± 0.0038
Physics	**0.9524 ± 0.0011**	0.9465 ± 0.0018	**0.8299 ± 0.0025**	0.8146 ± 0.0045

**Table 3:** Average performance in ARI and Jaccard Index metrics. The following table shows the comparison of ARI and Jaccard Index metrics on the model with and without the auxiliary learning branch. The experimental setup is consistent with the table above. The part in bold is the effect of the model proposed in this paper.

	ARI		Jaccard Index	
	NIGCN	GCN	NIGCN	GCN
Cora	**0.6674 ± 0.0124**	0.6410 ± 0.0039	**0.7043 ± 0.0086**	0.6878 ± 0.0035
CiteSeer	**0.4658 ± 0.0089**	0.4359 ± 0.0023	**0.5343 ± 0.0080**	0.5182 ± 0.0016
PubMed	**0.4450 ± 0.0026**	0.4271 ± 0.0036	**0.6426± 0.0015**	0.6322 ± 0.0020
CS	**0.8664 ± 0.0026**	0.8582 ± 0.0042	**0.7805 ± 0.0052**	0.7621 ± 0.0065
Physics	**0.8987 ± 0.0017**	0.8886 ± 0.0051	**0.8717 ± 0.0019**	0.8602 ± 0.0051

## Data Availability

The data presented in this study are available in GitHub at https://github.com/kimiyoung/planetoid and https://github.com/shchur/gnn-benchmark (accessed on 18 November 2025), reference number [18,19]. These data were derived from the following resources available in the public domain: Planetoid dataset (Cora, CiteSeer, PubMed) and Microsoft Academic Graph (Coauthor CS, Coauthor Physics).
